# Nutritional Educational Intervention in Users With Psychiatric Disorders Living in Supported Housing: A Pilot Study

**DOI:** 10.1016/j.focus.2025.100368

**Published:** 2025-05-16

**Authors:** Giulia Picardo, Clara Donnoli, Maria Rosaria Barbera, Marina Agostini, Giuseppe Ducci, Giuseppe Liotta

**Affiliations:** 1 Department of Biomedicine and Prevention, University of Rome Tor Vergata, Rome, Italy; 2 Department of Mental Health, ASL Rome 1, Rome, Italy

**Keywords:** Psychiatric disorder, lifestyle intervention, obesity, nutrition, education intervention

## Abstract

**Introduction:**

The pilot data analysis assessed the weight and eating habits of users in Supported Housing (SH) across six Rome districts, verifying the feasibility of a nutritional education intervention.

**Methods:**

The study team conducted a quantitative, quasi-experimental study. The cohort consisted of 92 users living in 57 houses, categorized by age, sex, and diagnosis. The intervention carried out between October 2021 and January 2022 consisted of 2 phases, an educational session on the food pyramid and national dietary guidelines for staff assisting users and an education intervention to promote healthier lifestyle choices to the users.

**Results:**

Results reported an average BMI of 28 kg/m^2^ (SD = ±5.74) categorizing users as belonging in average to the overweight BMI class. Adherence to the Mediterranean diet based on the Del Balzo and Savastano1 questionnaire was overall scarce (90.1%). The educational intervention positively impacted 90.3% of users who demonstrated an increase in consumption of fruit and vegetables and a decrease in consumption of fried food and soft drinks. A weight decrease was experienced by 43.4% (13/30) of the cohort. Average weight loss, in the group who lost weight, was 7.6kg (SD = ±6.93).

**Conclusions:**

The study suggests the feasibility and potential impact of public health initiatives addressing obesity in the population with psychiatric disorders.

## HIGHLIGHTS

• Nutritional education program was piloted in Supported Housing for patients with psychiatric disorders.
• Most participants were overweight (BMI=28 kg/m²) with poor adherence to the Mediterranean diet.
• Intervention increased fruit and vegetable intake and reduced fried food and soft drinks.
• Weight loss was observed in 43.4% of participants, averaging 7.6 kg in those who lost weight.
• Staff showed strong interest in continued nutrition education and specialist support.

## INTRODUCTION

Individuals with psychiatric disorders face aggravated physical health issues and reduced life expectancy, primarily owing to premature cardiovascular diseases.^1^, ^2^, ^3^ The intricate relationship between lifestyle factors, illness, and psychotropic medications contributes to this phenomenon.^4^ Individuals with severe mental disorders (SMDs) experience elevated mortality rates, primarily linked to noncommunicable diseases, including cardiovascular diseases, diabetes, and respiratory illnesses.^1^ Risk factors such as smoking and sedentary behavior further amplify these disparities.^4^ The increased prevalence of physical health conditions among those with SMDs is significant, with cardiovascular disease posing a 10-fold higher risk of death than suicide.^1^ In addition, individuals with SMDs exhibit higher rates of diabetes^2^ and are more susceptible to infectious diseases such as HIV/AIDS, tuberculosis, and hepatitis.^5^ Notably, individuals with schizophrenia face a fourfold increased risk of abdominal obesity^2^ and elevated rates of metabolic syndrome and hypertriglyceridemia.^2^ Psychotropic medications, including antipsychotics, are associated with metabolic side effects, emphasizing the need for balanced diets in treatment approaches.^6^, ^7^, ^8^, ^9^ The initiation of antipsychotic treatment, particularly with drugs such as clozapine and olanzapine, is linked to significant weight gain and unhealthy food preferences, elevating the risk of metabolic complications.^4^

Educational approaches and lifestyle modifications, including Mediterranean diet principles, have been shown to reduce weight in individuals with psychiatric disorders.^10^, ^11^, ^12^, ^13^, ^14^, ^15^, ^16^, ^17^, ^18^, ^19^, ^20^, ^21^ The intricate relationship between mental health, lifestyle, and physical well-being underscores the need for holistic approaches in psychiatric care. This holistic perspective is crucial for improving the overall quality of life and life expectancy of individuals with psychiatric disorders, especially in the context of the global burden of mental health disorders.

This pilot quasiexperimental study aims to identify the adherence of users with psychiatric disorders to a nutritional educational intervention and its effects on their diet and weight. Other outcomes include (1) determining the feasibility of the study protocol, (2) testing recruitment and consent rates, (3) piloting the measurement instrument (questionnaire), and (4) testing data entry and analysis.

## METHODS

This study is a pilot data analysis on the feasibility of a nutritional educational intervention for patients with severe mental health conditions living in supportive housing and for those who provide them health care. The protocol was approved by the local Ethics Committee of the Local Health Authority (LHA) RM 1 (N. 1251/CE Lazio 1), and all users provided written informed consent. The study was conducted in collaboration with the Mental Health Department of the LHA RM 1, which established the Supported Housing (SH) pathway in the Rome municipality. The intervention was performed between October 2021 and January 2022. Unlike other psychiatric outpatient services, which are often conducted in LHA-owned facilities, the SH program operates within users' private homes. This setting grants individuals a greater autonomy and limits the LHA's direct influence, ensuring that the educational intervention reflects real-life conditions in a noninstitutionalized environment.

The inclusion criteria were adults (aged >18 years, no upper limit) with SMDs defined according to DSM-5^22^ or International Classification of Diseases, Eleventh Revision criteria with the ability to give informed consent and participate in the SH program. The exclusion criteria included diagnosis of eating disorders, current diagnosis of an active substance–dependence disorder, residing in a nursing home or other institution, primary diagnosis of dementia or significant cognitive impairment, and history of violence.

The authors conducted a quantitative, quasiexperimental study, collecting data concurrently to inform future enhancements to the intervention model. Quantitative assessments of pre–post descriptive outcomes of weight were conducted at baseline and 3-month follow-up. In addition, qualitative data were collected through the administration of 3 questionnaires: Adherence to Mediterranean Diet by Del Balzo and Savastano,^23^ food frequency survey, and appreciation of the intervention survey. The JBI Checklist for Quasi-Experimental Studies^24^ was used to assess whether safeguards have been put in place to minimize the risk of bias or address other factors related to study validity or quality.

### Study Setting

**Housing model.** The program assists a total of 154 patients living in 95 apartments. All patients were invited to participate in the intervention on eating habits, but only a portion took part. Therefore, the setting of the study included 92 users, 28 females and 64 males, who lived in 57 houses. Patients were aged between 25 and 63 years. The homes hosted 1–4 users who lived together as roommates and received support from a multidisciplinary team of social and health workers. The paradigm around which the SH projects revolve is the person's biopsychosocial unity.^25^

On the basis of experience, the following conditions have been defined to allow inclusion and exclusion on the basis of specific eligibility criteria for access to the SH program:

1. Acceptance of the disease and the ability to live with symptoms without them impeding the possibility of living at home;
2. Collaboration with the assigned care team and accepting support in daily activities;
3. Acceptance of pharmacological therapy as an integral part of their life;
4. Having the ability to differentiate the urgency of questions and needs;
5. Ability to live in group contexts; and
6. Having the ability to learn daily administrative tasks.

The following are exclusion criteria from the SH pathway:

1. Abuse of psychotropic substances, including alcohol;
2. Dual diagnosis, defined as a mental illness and a comorbid substance use disorder;
3. Severe clinical decompensation;
4. The presence of severe organic pathologies;
5. The lack of consensus on pharmacotherapy; and
6. Forms of aggressiveness against themselves or others.

**Staff.** The SH pathway comprises a Clinical Manager, who is the Mental Health Department Director, and a Social Coordinator Assistant. The staff involved in the educational intervention included the following:

1. Social Assistant (1 coordinating each district), who fosters self-help relationships and aids in social security and administrative procedures;
2. Educator, who contributes significantly to individuals' well-being by facilitating relationships and providing support in self-care and home environment management; and
3. Socio Sanitary Operator, who supports individuals in self-care and managing their home environments.
4. Operators must have technical and professional competencies in addition to soft skills to deal with daily situations, respecting the user's freedom.^26^

### Interventions

The anthropometric values and sociodemographic characteristics of users who live in SH in 6 Roman districts were collected. Moreover, eating habits, smoking habits, and physical exercise frequency were noted. The participants completed a 25-item questionnaire. Responses were validated by comparing them with those of a 2-day food record collected by the operators. The questions were obtained from Adherence to Mediterranean Diet by Del Balzo and Savastano,^23^ a validated Italian instrument to evaluate adherence to the Mediterranean diet. To adapt the questionnaire to the population with psychiatric disorders, additional information, in parentheses, was added to further clarify the questions. The 2-day food record included questions about what the users ate during meals and snacks and outside the home. The participants reported a positive attitude toward the questionnaire.

Based on the results, the following 2 educational interventions—1 for the staff and 1 for the users—were organized:

1. For the staff of the SH program: an educational intervention on general principles of nutrition, including the food pyramid and the national guidelines for a healthy diet, was organized. The intervention was a 2-hour-long single session. It involved 30 participants and was followed by numerous questions on how to deal with specific situations and explanations of common myths about nutrition.
2. For the users: a nutritional education intervention was performed on a selected cohort of users to assess patient response. A cohort of 45 users was selected on the basis of overall health status and social skills. The intervention was a single, 1-hour-long session, with the participation of 1–15 individuals. The encounters were organized to provide tools to make better lifestyle choices and give information about the food pyramid, frequencies, and quantities of food categories. During the encounters, an example weekly menu plan, with recipes for each dish, was handed out to the participants. The menu had the main goal of increasing dietary variability, especially introducing new vegetables and less caloric ways to cook them. The effectiveness of the nutritional educational intervention was evaluated on the basis of a survey administered to participants to assess changes in eating habits. These included the changes in the quantities of vegetables, fruit, fried food, and soft drinks consumed and the attitude of the users toward meals and grocery shopping.

User weight, height, and BMI were evaluated at baseline and after 3 months to gain more insight. In addition, the operators evaluated the usefulness of the intervention and their ability to continue it over time, whereas the users described their interest in the intervention.

### Study Measures

Participant outcomes included diet modifications, weight loss, and BMI decrease. Weight was measured in kg on a flat, even surface using a calibrated digital scale in a pharmacy. Change in weight (kg) and BMI was calculated.

Participant satisfaction with the program was assessed using a de novo participant satisfaction questionnaire, which was designed to measure perceived satisfaction, usefulness, and ease of learning and tailored to the user characteristics (Appendix A, available online). This was used both for users and for SH staff.

### Statistical Analysis

Descriptive and inferential statistical analyses were performed with the SPSS software, Version 26. Preliminary descriptive and confirmatory analyses were conducted, such as bivariate and multivariate analyses. The main statistical tests used were parametric and nonparametric. Normality was tested using the Shapiro–Wilk test. For normally distributed variables, comparisons between group means were made using *t*-test, whereas for non-normally distributed variables, the Mann–Whitney *U* test was applied. A 1-way ANOVA (Kruskal–Wallis test) was also applied for the comparison among more than 2 non-normally distributed variables.

## RESULTS

Preliminary analyses were performed to verify the distribution of the data set variables. Weight and BMI appeared normally distributed, whereas the score for the adherence to the Mediterranean diet questionnaire did not appear normally distributed, as assessed by Shapiro–Wilk test. Therefore, both parametric and nonparametric analyses were used for these data, on the basis of the distribution of the variables.

The reference population of 92 users living in the SH protocol is made up of 53 (57.6%) males and 39 (42.4%) females. The mean (SD) age was 56.1 (10.4) years, 58.1 years for females and 54.8 years for males (Table 1). The highest number of users (34.4%) was in the age group of 45–54 years.

**Table 1:** Anthropometric and Sociodemographic Characteristics of the Intervention Group and Nonintervention Group

Characteristic	Group	*n*	Missing	Mean (SD)	Median	Range (minimum–maximum)
Age, years	Intervention	67	0	53.6 (17.1)	54	19–86
	Nonintervention	23	0	53.0 (18.4)	54	21–88
Height (cm)	Intervention	67	0	172.3 (9.2)	172	155–195
	Nonintervention	23	0	171.4 (9.8)	170	158–190
Weight T0 (kg)	Intervention	67	0	82.2 (14.9)	80	55–114
	Nonintervention	23	0	81.7 (16.1)	80	56–115
Weight T1 (kg)	Intervention	67	0	79.3 (14.8)	77	53–110
	Nonintervention	0	23	—	—	—
BMI	Intervention	67	0	27.6 (4.6)	27	18.2–41.2
	Nonintervention	23	0	27.7 (4.7)	27.2	19.0–39.5
Test score	Intervention	67	0	2.6 (1.4)	3	1–5
	Nonintervention	23	0	2.6 (1.3)	2	1–5
Weight difference (kg)	Intervention	67	0	−2.9 (3.9)	−2	−12, 8
	Nonintervention	0	23	—	—	—

The educational level was classified into 3 groups: 43.3% (39 of 90) people had a lower education level (elementary or middle school), 51.1% (46 of 90) had intermediate level (high school or professional school), and 5.6% (5 of 90) had higher level (university degree). The cohort was composed of 52.7% smokers and 47.3% nonsmokers.

The user’s background can be categorized into the following 4 groups: residential psychiatric structures, family, home alone, and condition of homelessness.

**Figure 1:**
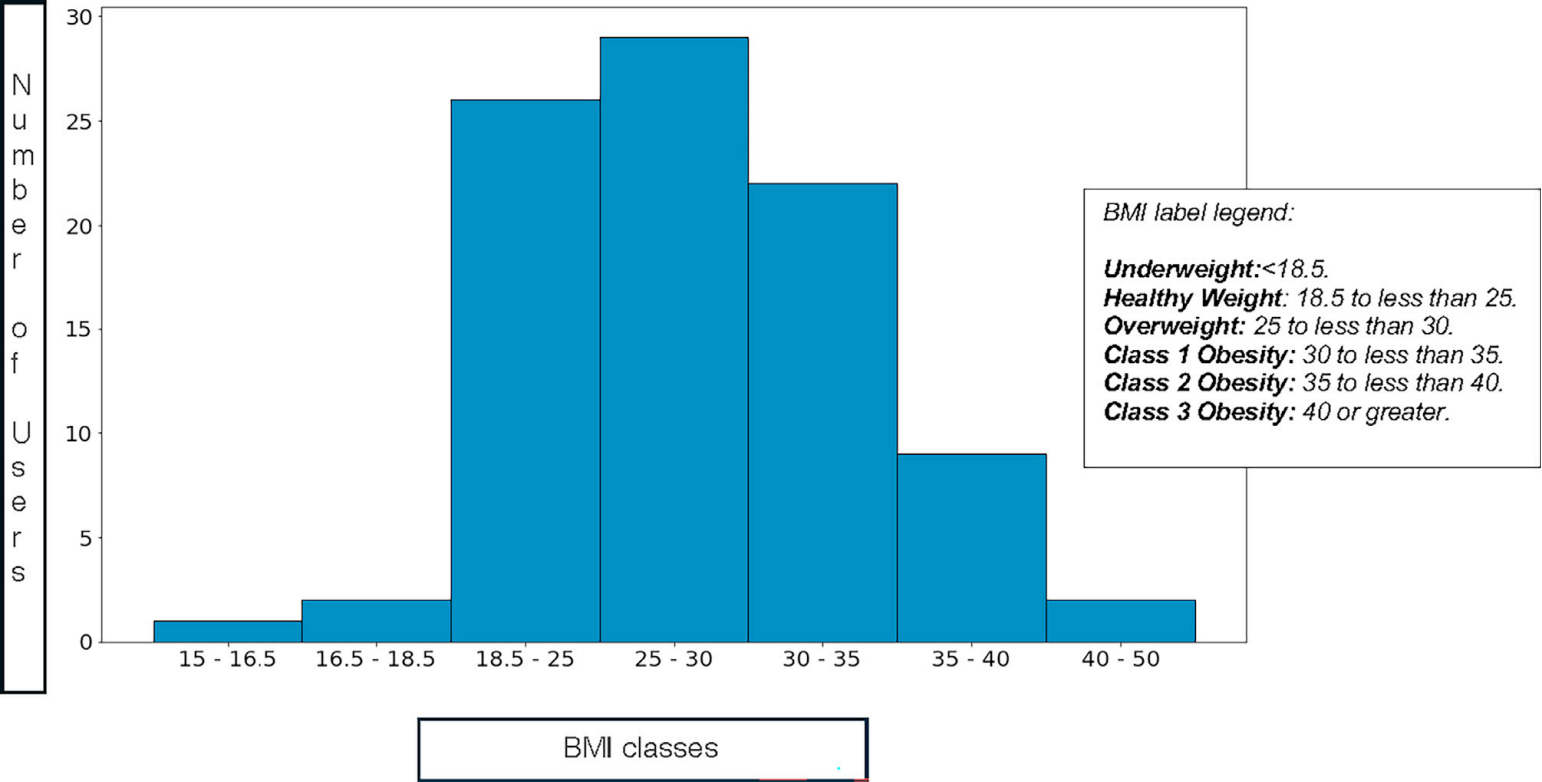
BMI distribution.

The participants mean (SD) weight was 80.3 (19) kg, specifically 73.2 (17.7) kg for females and 85.4 (18.3) kg for males. The average (SD) BMI was 28 (5.74) kg/m^2^, ranging from 16.2 kg/m^2^ to 42.1 kg/m^2^, with an average (SD) BMI of 27.9 (6.31) kg/m^2^ for females and 29.1 (5.36) kg/m^2^ for males. In the cohort, 67.4% (61 of 91) of the users had a BMI >25 kg/m^2^, considered overweight, whereas only 29% (26 of 91) were normal weight, and 3.5% (3 of 91) were underweight. Of the overweight users, 44.8% (27 of 61) were preobese, 36.2% (22 of 61) were Class I obese, 15.5% (9 of 61) were Class II obese, and 3.4% (2 of 61) were Class III obese (Figure 1). An independent *t*-test was performed between male and female BMI, which resulted in no statistical significance.

The Adherence to Mediterranean Diet questionnaire^1^ was administered to the sample. Adherence values to the Mediterranean diet were considered scarce if the score was ≤5, medium if it was 5–9, and good if it was ≥10. The questionnaire comprises 14 items that assess adherence to a Mediterranean diet by evaluating daily and weekly consumption of various food groups. It assigns points on the basis of intake thresholds for olive oil, vegetables, fruits, legumes, fish, nuts, and whole grains. It also considers the consumption frequency of unhealthy foods such as red meat, processed meat, butter, and sugary drinks. A higher score indicates better compliance with Mediterranean diet patterns. The total score helps to classify individuals on the basis of their diet quality and potential health benefits.

**Figure 2:**
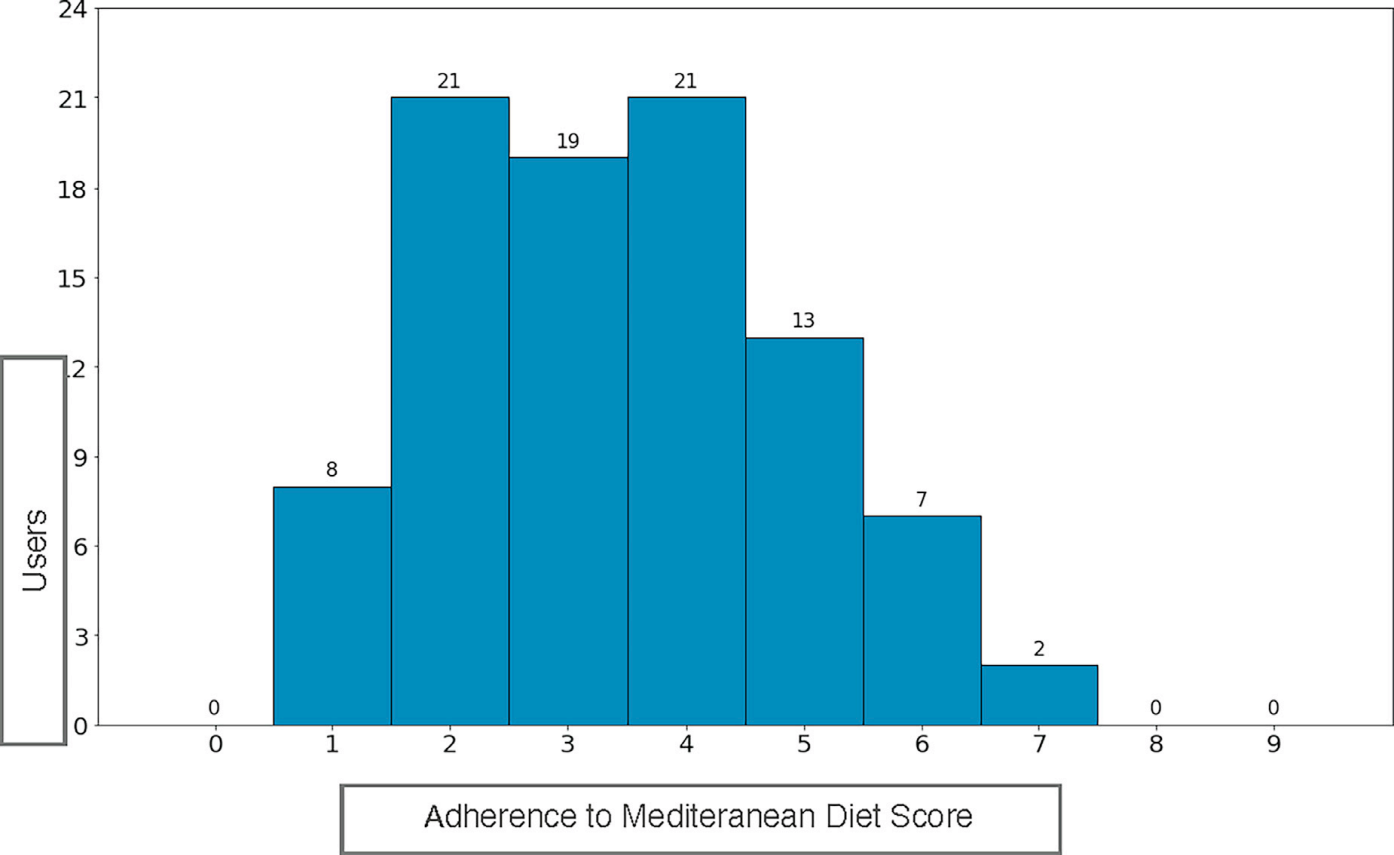
Adherence to Mediterranean diet score.

All the users scored ≤7, specifically 90.1% earned a score ≤5, indicating a scarce adherence to the Mediterranean diet, and 9.9% had a score between 5 and 9, indicating medium adherence (Figure 2), suggesting that the obesity observed in the sample was highly dependent on the type of diet. Females demonstrated a statistically significant higher score to the questionnaire than males (Mann–Whitney *U*=763, *p*=0.04; 95% CI=0.01, 1). A 1-way ANOVA (Kruskal–Wallis test) was conducted to compare the score between 3 different education levels (higher, middle, and lower). Considering that most of the participants were at the lower and middle educational levels, only these results were taken into account. The users in the middle education group obtained a significantly higher score than the users in the lower education group (Kruskal–Wallis test *p*=0.039).

Baseline mean (SD) weight was 80.3 (19) kg, 73.2 (17.7) kg for females and 85.4 (18.3) kg for males. The average (SD) BMI was 28 (5.74) kg/m^2^, ranging from 16.2 kg/m^2^ to 42.1 kg/m^2^, with an average BMI (SD) of 27.9 (6.31) kg/m^2^ for females and 29.1 (5.36) kg/m^2^ for males.

In the 3-month period, weight loss was observed in 43.3% (13 of 30) of the participants who took part in the educational sessions, 22% (2 of 9) of females and 52.3% (8 of 21) of males. One male and 2 females maintained their initial weight. The average (SD) weight loss in the group who lost weight was 7.6 (6.93) kg. Fourteen users were lost to follow up owing to the difficulty in maintaining a connection between the LHA and external cooperatives providing support staff.

Nutritional improvements were observed on the basis of qualitative assessments. A total of 90.3% of the participants demonstrated greater mindfulness around eating, as reported by support staff. Dietary changes included increased vegetable intake (74.1%), increased fruit consumption (58%), reduced sweets and soft drinks (64.5%), and a decline in fried food consumption (70.9%).

The intervention was well received, with 85.7% of the staff (26 of 30) finding it useful and 97.1% (29 of 30) appreciating the teaching methodology. In addition, 85.7% (26 of 30) supported continuing the intervention, whereas 94.2% (28 of 30) expressed a need for further support.

## DISCUSSION

The objective of this pilot study was to evaluate the feasibility of a nutritional educational intervention for individuals with SMDs. The results of this early-stage pilot data analysis suggest that the principles of healthy eating provide a useful starting point to promote weight loss and weight management in people with SMDs. The data collected in this pilot study are in line with the reports of the elevated percentage of obesity in individuals with mental disorders.^27^ Improving food choices, restricting portion size, and increasing physical activity are essential to weight-loss programs.^28^ However, achieving these goals with users who have SMDs poses challenges, including difficulty in motivating them to continue in organized programs, limited financial support, and the adverse metabolic effects of psychotropic medication.^7^^,^^29^^,^^30^ Despite these challenges, the results, in accordance with those of other authors,^17^^,^^19^^,^^20^^,^^31^^,^^32^ dispel the belief that users with chronic psychiatric conditions are not able, because of mental and physical limitations, to maintain consistent participation in a weight-loss program. It is important to note that the participants were clinically stable and motivated to participate by the staff.

The study was performed in a large urban area, encompassing diverse economic, social, and family backgrounds; in addition, the users had varying educational levels, self-sufficiency, and mental disabilities related to the diagnosis of SMDs, age, and previous experiences. The analysis supports the concept that higher educational levels are associated with better adherence to the Mediterranean diet. This is reflected in the higher scores among individuals with higher education levels and among females, who are historically more involved in home care and cooking.^33^^,^^34^ The data strongly suggest that there is a need for public health management activities that focus on the prevention of obesity and metabolic disorders in the community-dwelling individuals with psychiatric disorders, aimed at behavioral lifestyle interventions and helping to put these suggestions into practice.^35^

The results show that the collaboration between social service operators and a nutrition expert is an approach with promising results. The introduction of a nutrition expert sparked user interest and, with the operators' involvement, enhanced the continuity and effectiveness of the intervention. The satisfaction evaluation showed that the operators had positive opinions, demonstrating awareness of the issue and methodology. They also showed an interest in being supported over time, suggesting organized encounters every 3 months between the operators and a nutrition specialist to gain further insight, address possible issues, and modify the menus on the basis of seasonality. The strength of this study is the organization of a nutrition educational intervention in an LHA setting that involved collaboration with public employees to enhance their knowledge on the topic. To the authors' knowledge, there are only a few other studies that have conducted this type of research in real-world settings.^20^^,^^36^

### Limitations

Limitations of the study include a small cohort, varying in numerous aspects, with a selection of the study group that is not representative of the general population of the SMD community. In addition, the short follow-up period did not yield significant modifications in weight, BMI, and metabolic parameters. Patient progress was strongly influenced by the support and motivation received from the supporting professionals. Further studies could also consider longer study periods and markers such as body composition, metabolic parameters, and aerobic exercise capacity.

## CONCLUSIONS

The intervention proved beneficial, encouraging the inclusion of a nutrition expert to improve users' quality of life and metabolic health, which is closely linked to their psychiatric condition. Despite the difference in the results found in the cohort, the most crucial factor to consider is the positive attitude of users toward the intervention and the significant increase in awareness of nutritional habits. It is advisable to involve users with SMDs in nutritional education aimed at developing appropriate dietary skills.

## Appendix Supplementary materials

Image, application 1

## Footnotes

Supplementary material associated with this article can be found in the online version at doi:10.1016/j.focus.2025.100368.
