# Supplementary material for: Nutritional Educational Intervention in Users With Psychiatric Disorders Living in Supported Housing: A Pilot Study
Source: AJPM Focus. 2025 May 16;4(6):100368. doi: 10.1016/j.focus.2025.100368 (PMC12480872; doi:10.1016/j.focus.2025.100368)
Supplement: Supplementary file 1 [file mmc1.docx]

Appendix A

**Evaluation of the Intervention Based on Patient Behavior**

| \| **Question** \| **Rating (1-5)** \| \| --- \| --- \| \| Has the patient increased fruit consumption? \|  \| \| Has the patient increased vegetable consumption? \|  \| \| Has the patient reduced sweet consumption? \|  \| \| Has the patient reduced sugary drink consumption? \|  \| \| Has the patient reduced fried food consumption? \|  \| \| Has the patient increased nutritional awareness? \|  \| \| Has the patient adhered to the proposed weekly menu? \|  \| \| Satisfaction with the intervention \|  \| \| Does the patient receive additional household support? \|  \| \| **Weight:** __________ \| **Date:** __________ \| |
| --- | --- | --- | --- | --- | --- | --- | --- | --- | --- | --- | --- | --- | --- | --- | --- | --- | --- | --- | --- | --- | --- | --- |

**Evaluation of the Intervention Based on the Operator’s Opinion**

| \| **Anonymous Questionnaire – Usefulness of the Nutrition Intervention for Operators** \| **Rating (1-5)** \| \| --- \| --- \| \| How useful was the training session? *(How often were the learned concepts applicable?)* \|  \| \| How well was the training session received? *(Was the topic worth the time spent?)* \|  \| \| To what extent do you feel capable of continuing the intervention with the skills acquired? \|  \| \| To what extent do you think there is a need for prolonged nutritional support? \|  \| |
| --- | --- | --- | --- | --- | --- | --- | --- | --- | --- | --- |
